# *Ilex latifolia* Improves the Anti-Tumor Effectiveness of Rapamycin Against Breast Cancer In Vitro and In Vivo

**DOI:** 10.3390/foods14091477

**Published:** 2025-04-24

**Authors:** Zhengnan Ren, Yikuan Wu, Xiaoying Guo, Haizhi Tian, Hongjing Ou, Zihan Xiong, Yu Xiao, Longquan Xiao, Jing Li, Haibo Wu, Xinhui Wang

**Affiliations:** 1 College of Food and Biological Engineering, Chengdu University, Chengdu 610106, China; rzhengnan@outlook.com (Z.R.);; 2 Institute of Science and Technology, Jiangnan University, Wuxi 214122, China; 3 Sichuan Kelun Pharmaceutical Co., Ltd., Chengdu 610599, China; 4 School of Food Science and Technology, Jiangnan University, Wuxi 214122, China; 5 Turpan Institute of Agricultural Sciences, Xinjiang Academy of Agricultural Sciences, Turpan 838099, China

**Keywords:** *Ilex latifolia*, rapamycin, breast cancer, dietary intervention

## Abstract

Breast cancer remains one of the leading causes of cancer-related mortality among women worldwide. Although the mTOR inhibitor rapamycin exhibits notable anti-tumor activity, its clinical application is limited by metabolic side effects, particularly dyslipidemia. This study aimed to investigate the potential of *Ilex latifolia* (*I. latifolia*, large-leaf kudingcha), a traditional Chinese tea known for its lipid-lowering properties, to enhance the therapeutic efficacy of rapamycin in breast cancer. The combined effects of *I. latifolia* and low-dose rapamycin on tumor cell proliferation, cell cycle progression, apoptosis, and inflammation were assessed in four breast cancer cell lines and a murine breast cancer model. While low-dose *I. latifolia* alone exhibited limited anti-tumor activity, its combination with low-dose rapamycin synergistically inhibited tumor proliferation, induced cell cycle arrest, promoted apoptosis, and reduced inflammation in vitro. In vivo, dietary supplementation with *I. latifolia* mitigated rapamycin-induced lipid disturbances, reduced tumor growth, enhanced apoptosis, and alleviated inflammation in tumor tissues. These findings highlight *I. latifolia* as a promising dietary adjunct to rapamycin, providing a safer and more effective combinatorial strategy for breast cancer treatment.

## 1. Introduction

Breast cancer ranks among the most prevalent cancers in women worldwide and continues to be a primary contributor to cancer-related deaths [1]. Early detection and advanced treatment strategies, such as surgery, chemotherapy, radiation therapy, and targeted therapies, have significantly improved survival rates [2]. However, recurrence and metastasis continue to present major challenges, particularly in advanced and metastatic cases [2]. The heterogeneity of breast cancer, with various subtypes such as triple-negative breast cancer (TNBC) and human epidermal growth factor receptor 2 (HER2)-positive breast cancer, further complicates therapeutic approaches [3,4]. These variations make applying a one-size-fits-all treatment strategy difficult, necessitating the development of novel therapeutic interventions. In recent years, natural products have garnered increasing interest in cancer research due to their multi-target mechanisms and relatively low toxicity compared to synthetic drugs [5,6]. These compounds often offer complementary therapeutic effects and are being explored as potential adjuvants in cancer treatment.

Rapamycin, a well-known mammalian target of rapamycin (mTOR) inhibitor, has demonstrated considerable anti-cancer potential, particularly in inhibiting tumor cell proliferation, blocking angiogenesis, and inducing autophagy [7]. However, as a single agent, the clinical effectiveness of rapamycin in cancer treatment is limited [8]. Prolonged use of rapamycin can lead to significant side effects, including hyperlipidemia, metabolic disorders, and the emergence of drug resistance [8]. Furthermore, the immunosuppressive effects of rapamycin can increase the risk of infections and compromise overall patient health [9]. These limitations have prompted researchers to explore combination therapies to enhance their therapeutic potential. While several studies have focused on combining rapamycin with synthetic drugs [10,11,12], there is limited research on using natural food components or herbal products to enhance the anti-tumor effects of rapamycin.

Kudingcha is a traditional herbal tea widely consumed in China and comprises two primary species: *Ilex latifolia* (*I. latifolia*, large-leaf kudingcha) and *Ligustrum robustum* (*L. robustum*, small-leaf kudingcha). Ethnopharmacological and ethnobotanical studies have shown that both *I. latifolia* and *L. robustum* have long been used in traditional medicine for their anti-inflammatory, antioxidant, and lipid-lowering properties [13,14,15,16,17,18]. Interestingly, previous studies further demonstrated the potential of *I. latifolia* to inhibit tumor cell proliferation, promote apoptosis, and modulate the tumor immune microenvironment [19,20]. However, its pronounced bitterness may limit its acceptability for long-term or high-dose consumption [13]. Given its natural origin, favorable safety profile, and lipid-lowering effects, *I. latifolia* holds promise as a dietary adjunct to rapamycin, potentially enhancing anti-tumor efficacy while mitigating its metabolic side effects.

This study aims to evaluate the adjuvant potential of *I. latifolia* in combination with low-dose rapamycin for breast cancer treatment, using both in vitro and in vivo models. Interestingly, our study has identified a non-toxic dose of *I. latifolia* that does not directly inhibit breast cancer cell growth. However, this dose significantly improves the anti-tumor efficacy of rapamycin. This combination therapy may provide a promising and less-toxic alternative for the treatment of breast cancer, potentially improving patient outcomes without exacerbating side effects.

## 2. Materials and Methods

### 2.1. Preparation of I. latifolia Extracts

*I. latifolia* and *L. robustum* were purchased from Efuton Tea Co., Ltd. (Zhejiang, China). The dried tea was steeped in distilled water (1 g tea: 10 mL water) for 30 min, followed by three cycles of extraction. In each cycle, the mixture was heated in a water bath at 100 °C for 1 h, and the supernatant was collected. After each extraction, 10 mL of fresh distilled water was added to the residue, and the process was repeated. The extracts from all three cycles were combined, filtered, and concentrated to one-third of the original volume using a rotary evaporator under reduced pressure. The final extract was sterilized and adjusted to 0.1 g/mL and then stored at −20 °C until further use.

### 2.2. Characterization of I. latifolia Extracts

Five-tenths milliliter of the *I. latifolia* extracts was incorporated into a combination of chromatographic-grade methanol and ultrapure water (2:1, *v*/*v*), thereafter subjected to vortex mixing for 2 min and ultrasonic treatment for 5 min. The solution was subsequently filtered using a 0.22 μm membrane, and 1.5 mL of the filtrate was placed into a chromatography vial for analysis. The liquid chromatography analysis utilized the ACQUITY UPLC H-Class (Waters, MA, USA), employing mobile phases of 0.1% formic acid in water (Phase A) and 0.1% formic acid in acetonitrile (Phase B). The column was the BEH C18 (Waters, 1.7 μm, 2.1 × 50 mm). Mass spectrometry was conducted utilizing the G2-XS QTof MS instrument (Waters), employing both positive and negative ion modes. All organic reagents were purchased from Sinopharm Chemical Reagent Co., Ltd., (Shanghai, China). The gathered data were analyzed utilizing MassLynx 4.2 software (Waters), and secondary mass spectrometry (MS/MS) fragmentation peaks were first compared with the UNIFI natural product database, followed by a comparison with literature data or standard references for identification.

### 2.3. Cell Culture

The human breast cancer cell lines HCC1806, MDA-MB-231, MCF-7, and SK-BR-3 (ATCC, VA, USA), representing different molecular subtypes, were cultured under specified conditions. HCC1806 and MDA-MB-231 are TNBC cell lines characterized by the absence of estrogen receptor (ER), progesterone receptor (PR), and human epidermal growth factor receptor 2 (HER2) [21]. In contrast, MCF-7 cells are luminal A subtype, positive for ER and PR, while SK-BR-3 cells belong to the HER2-positive subtype, overexpressing HER2 [22]. HCC1806 cells were maintained in RPMI 1640 medium (Gibco^TM^, Grand Island, NY, USA) with 10% fetal bovine serum (Gibco^TM^) and 1% penicillin–streptomycin (Gibco^TM^). MDA-MB-231, MCF-7, and SK-BR-3 cells were grown in high-glucose Dulbecco’s Modified Eagle Medium (DMEM, Gibco^TM^) with 10% fetal bovine serum and 1% penicillin–streptomycin. MCF-10A cells, a non-tumorigenic, immortalized human mammary epithelial cell line, were used as the negative control (ATCC, VA, USA). These cells were cultured in DMEM supplemented with 5% horse serum (Gibco^TM^), 20 ng/mL epidermal growth factor (Gibco^TM^), 0.5 µg/mL hydrocortisone (Merck, Shanghai, China), 100 ng/mL cholera toxin (Merck), and 10 µg/mL insulin (Merck). All cell lines were incubated at 37 °C in a humidified atmosphere containing 5% CO_2_. Cell handling and all related procedures were conducted under sterile conditions in a designated biosafety cabinet. Sub-culturing was performed using 0.25% trypsin–EDTA (Gibco^TM^) when cell confluence reached approximately 80–90%.

### 2.4. Cell Treatment

After cell adhesion, different concentrations of single and combined treatments were applied. Each treatment group included three replicates. The concentration gradient of tea water extract was 0, 0.125, 0.25, 0.375, and 0.5 mg/mL. The stock solution was diluted with anhydrous ethanol to achieve the final working concentrations before application. The concentration gradient of rapamycin (MedChemExpress LLC, Shanghai, China) was 0, 2, 4, 8, and 16 μmol/L [23]. The combination treatment (combined group) was administered with 0.25 mg/mL tea water extract and 8 μmol/L rapamycin.

### 2.5. Cell Counting Assay

Cells in the logarithmic growth phase were harvested and resuspended, and the cell suspension was adjusted to a concentration of 2.0 × 10^5^ cells/mL. The suspension was then seeded into 6-well plates, with 2 mL of cell suspension per well. After incubating for 12 h in a cell culture incubator, the cells adhered to the surface of the wells. Following the treatment protocol, the cells were exposed to the appropriate drugs at the specified concentrations. After 72 h of continued incubation, the old medium was removed, and the cells were washed three times with 1–2 mL of PBS. Then, 200 μL of trypsin-EDTA solution was added for cell digestion, following standard procedures as previously described. The digestion was terminated by adding 800 μL of complete growth medium. The cell suspension was carefully pipetted to achieve single-cell dispersion. The cell suspension was transferred to a 1.5 mL tube, and 10 μL of the suspension was placed on a hemocytometer. Cell counts were conducted using an inverted microscope.

### 2.6. CCK-8 Cell Cytotoxicity Assay

Cells in the logarithmic growth phase were harvested and resuspended, and the concentration of the cell suspension was adjusted to 0.5 × 10^5^–1.0 × 10^5^ cells/mL. Using a multi-channel pipette, 100 μL of the suspension (approximately 1.0 × 10^4^ cells) was seeded into each well of a 96-well plate. To minimize errors caused by evaporation, 100 μL of PBS was added to the wells along the edges of the plate. The plate was incubated in a cell culture incubator for 12 h to allow cell adhesion. Following the experimental protocol, the cells were treated with the designated drugs and concentrations. After 72 h of incubation, the old medium was aspirated, and 100 μL of fresh complete growth medium, along with 10 μL of CCK-8 solution, was added to each well. The plate underwent incubation for an additional 2 h. The optical density at 450 nm was measured using a microplate reader. The cell death rate is calculated using the formula provided by the manufacturer of the CCK-8 kit (Dojindo Laboratories Co., Ltd., Kumamoto, Japan).

### 2.7. Cell Cycle Analysis

During the logarithmic growth phase, cells were harvested and resuspended to a concentration of 2.0 × 10^5^ cells/mL. The cell suspension was then evenly seeded into 6-well plates, with 2 mL per well. After incubating for 12 h in a cell culture incubator at 37 °C and 5% CO_2_ to facilitate cell adhesion, the cells were subsequently treated with the designated drugs at specified concentrations, as described in the experimental setup. After 24 h of incubation, cells were collected and resuspended in 300 μL of PBS (pre-cooled at 4 °C overnight). The suspension was transferred into 1.5 mL microcentrifuge tubes, and 700 μL of anhydrous ethanol (pre-cooled at −20 °C overnight) was added dropwise for fixation at 4 °C overnight. After centrifugation, the ethanol was eliminated, and the cells were subsequently washed once with PBS. The cells were then resuspended in 50 μg/mL propidium iodide staining solution containing 0.1% Triton X-100 and 100 μg/mL RNase (Servicebio, Hubei, China). The mixture was incubated in the dark at room temperature for 15 min. Finally, cell cycle distribution was analyzed using a flow cytometer (Attune^®^ NxT, Thermo Fisher Scientific, Waltham, MA, USA), and the data were processed using Flowjo software (v 10.8.1).

### 2.8. Western Blot

The cells underwent two washes with pre-cooled PBS and were subsequently lysed on ice utilizing the RIPA buffer that contained protease and phosphatase inhibitors. (Beyotime, Shanghai, China). The lysates were centrifuged at 12,000× *g* for 15 min at 4 °C, and the supernatants were collected. Protein concentrations were determined using the BCA protein assay kit (Beyotime), and equal amounts of protein (30–50 μg) were loaded onto SDS-PAGE gels for separation. After electrophoresis, proteins were transferred to PVDF membranes (Merck), which were blocked with 5% non-fat milk in TBST for 1 h at room temperature. The membranes underwent overnight incubation at 4 °C with primary antibodies specific for p21 (27296-1-AP, Proteintech, Chicago, IL, USA), p27 (26714-1-AP, Proteintech), and β-actin (20536-1-AP, Proteintech, used as a loading control). After washing the membranes with TBST, they were incubated with HRP-conjugated secondary antibodies for 2 h at room temperature (Thermo Fisher Scientific). The membranes underwent rewashing with TBST, and protein bands were visualized utilizing an enhanced chemiluminescence (ECL) detection system (ProteinSimple, San Jose, CA, USA). The intensity of the protein bands was quantified using ImageJ software (1.54g), and the relative expression levels of p21 and p27 were normalized to β-actin. Each experiment was performed in triplicate to ensure reproducibility.

### 2.9. Quantitative PCR Analysis

RNA extraction was performed on treated cells and mouse tumor tissues utilizing TRIzol reagent. (Thermo Fisher Scientific), followed by reverse transcription into cDNA. Gene expression levels were analyzed via qPCR using specific primers (Table 1). *ACTB* and *Actb* were used as internal controls in the cell and animal experiments, respectively. The expression levels of target genes were determined using the 2^−ΔΔCt^ method.

### 2.10. Experimental Animals and Treatment

Six-week-old BALB/c nude mice (GemPharmatech, Jiangsu, China) were housed under controlled conditions (22–25 °C, 12 h light/dark cycle) with free access to food and water (Jiangnan University, Jiangsu, China). Following a one-week adaptation period, mice were randomly assigned to four groups. (*n* = 5 per group). HCC1806 cells were resuspended in serum-free medium to 2.0 × 10^7^ cells/mL and mixed with Matrigel (Corning, NY, USA). Subcutaneous injection of 100 μL of the cell–Matrigel mixture was administered to each mouse. Once tumors reached about 200 mm^3^, the groups were fed the respective diet. The *I. latifolia* water extract was filtered and added to the normal maintenance feed at a ratio of 5 g of dried tea per kg of feed powder, which was then pelleted. Rapamycin was added to the feed at a dosage of 20 mg/kg feed. Tumor size was assessed every four days, and after 16 days, serum and tumor tissues were collected for subsequent analysis. All animal procedures adhered to the guidelines established by the Institutional Animal Ethics Committee of Jiangnan University (JN. No20170213-20171221[6]), and the animal studies followed the ARRIVE reporting guidelines [24].

### 2.11. Immunohistochemistry of Tumor Tissue

Tumor tissues were preserved in 4% paraformaldehyde and subsequently embedded in paraffin. Sections measuring 5 µm were prepared and affixed to slides. Antigen retrieval was conducted by heating the sections in citrate buffer (pH 6.0) at 95 °C for 30 min. Following a 5% BSA blocking step, the sections were incubated overnight at 4 °C with primary antibody anti-Ki67 (28074-1-AP, Proteintech). After PBS washes, slices were incubated with HRP-conjugated secondary antibody for 1 h at room temperature. The signals were visualized with 3,3′-diaminobenzidine, and nuclei were counterstained with hematoxylin. The stained sections were examined under a light microscope, and Ki67-positive cells were quantified.

### 2.12. Data Analysis

All data are expressed as the mean ± standard deviation (SD). Statistical analysis was conducted utilizing GraphPad Prism (version 9.5.0). A one-way analysis of variance (ANOVA) accompanied by Tukey’s post hoc test was employed for comparisons among various groups. Differences with *p* < 0.05 were deemed statistically significant. All experiments were conducted a minimum of three times to guarantee repeatability.

## 3. Results

### 3.1. The Main Components of Ilex latifolia Extract Are Triterpene Saponins and Phenolic Acid

The chemical composition of *I. latifolia* extract was characterized using Fourier-transform infrared spectroscopy, ultra-performance liquid chromatography coupled with quadrupole time-of-flight mass spectrometry, and the UNIFI natural product database. Phytochemical analysis revealed a complex profile, primarily consisting of polyphenolic and saponin compounds, which together accounted for over 92% of the identified components. Secondary metabolites included alkaloids and terpenoids, while organic acids and lipids were present in smaller amounts. Trace quantities of specialized metabolites, such as aromatic compounds, carbohydrates, steroids, glycosides, amino acids, ketones, and alcohols, were also detected (Table 2). Mass spectrometry data were processed using the UNIFI platform and manually validated. The predominant components identified in the extract were 1,4-dicaffeoylquinic acid, 1-caffeoylquinic acid, esculentoside L, ruberythric acid, and cynanoside O (Table 3).

### 3.2. Ilex latifolia, Ligustrum robustum, and Rapamycin Inhibit Breast Cancer Cell Proliferation in a Way Dependent on Dosage

**Figure 1:**
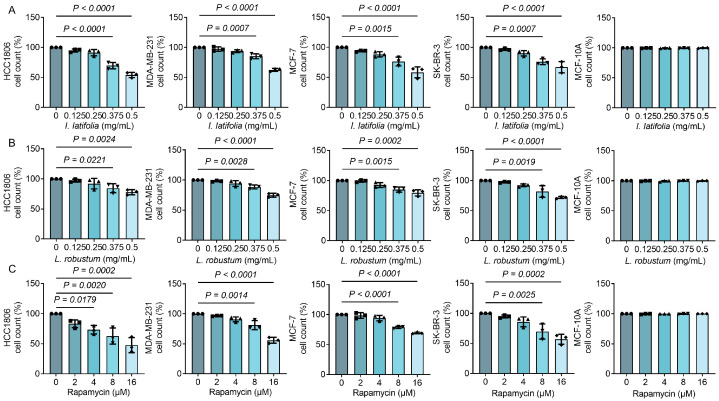
The inhibitory effects of *Ilex latifolia*, *Ligustrum robustum*, and rapamycin on breast cancer cells. (**A**) Percentage of cell count under different concentrations (0, 0.125, 0.25, 0.375, and 0.5 mg/mL) of *I. latifolia* treatment in HCC1806, MDA-MB-231, MCF-7, SK-BR-3, and MCF-10A cells. (**B**) Percentage of cell count under different concentrations (0, 0.125, 0.25, 0.375, and 0.5 mg/mL) of *L. robustum* treatment in HCC1806, MDA-MB-231, MCF-7, SK-BR-3, and MCF-10A cells. (**C**) Percentage of cell count under different concentrations (0, 2, 4, 8, and 16 μM) of rapamycin treatment in HCC1806, MDA-MB-231, MCF-7, SK-BR-3, and MCF-10A cells. The initial cell count was set to 100%. Data in (**A**–**C**) were presented as mean ± SD from three independent experiments. *p* values in (**A**–**C**) were determined by Tukey’s multiple comparisons test following one-way ANOVA. *p* < 0.05 was considered statistically significant. Triangles, diamonds, circles, and squares are used solely to differentiate between experimental groups and do not indicate specific biological meanings. Each dot represents data from an independent experiment.

To evaluate the anti-tumor effects of *I. latifolia*, *L. robustum*, and rapamycin, we selected four representative breast cancer cell lines: HCC1806 and MDA-MB-231 (triple-negative breast cancer, characterized by high invasiveness and metastatic potential), MCF-7 (estrogen receptor-positive breast cancer, representing hormone-dependent tumors), and SK-BR-3 (HER2-overexpressing breast cancer). These cell lines encompass the major molecular subtypes of breast cancer, reflecting the diversity of breast cancer classifications. The findings indicated that both *I. latifolia* and *L. robustum* displayed substantial inhibitory effects on the proliferation of all four cell lines at concentrations of 0.375 and 0.5 mg/mL, respectively (Figure 1A,B). Rapamycin markedly decreased cell counts across all four cell lines at concentrations of 8 and 16 μM (Figure 1C). Notably, no inhibitory effects were observed on the MCF-10A cells, a normal mammary epithelial cell line, suggesting that these compounds selectively target cancer cells without affecting normal breast cells. These findings highlight the dose-dependent inhibitory effects of *I. latifolia*, *L. robustum*, and rapamycin on breast cancer cell proliferation.

### 3.3. Ilex latifolia Amplifies the Antiproliferative Effect of Rapamycin on Breast Cancer Cells

**Figure 2:**
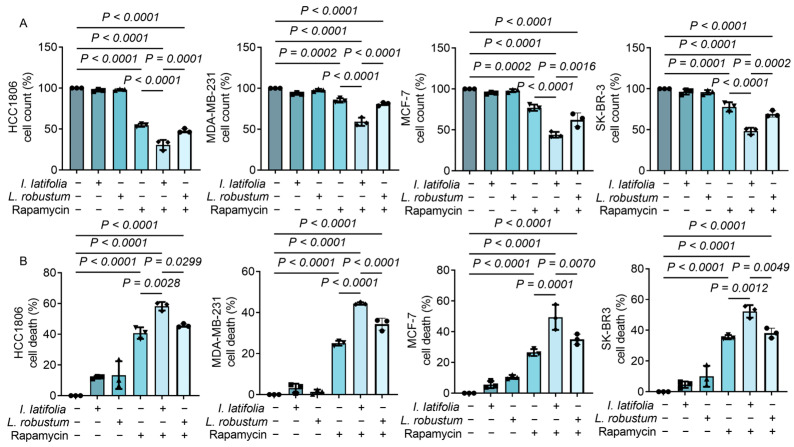
Effect of *Ilex latifolia* or *Ligustrum robustum* combined with rapamycin on breast cancer cells. (**A**) Percentage of cell count after treatment with *I. latifolia* (0.25 mg/mL), *L. robustum* (0.25 mg/mL), rapamycin (8 μM), *I. latifolia* + rapamycin, and *L. robustum* + rapamycin in HCC1806, MDA-MB-231, MCF-7, and SK-BR-3 cells. The initial cell count was set to 100%. (**B**) Percentage of cell death following the same treatments in HCC1806, MDA-MB-231, MCF-7, and SK-BR-3 cells. Data in (**A**,**B**) were presented as mean ± SD from three independent experiments. *p* values in (**A**,**B**) were determined by Tukey’s multiple comparisons test following one-way ANOVA. *p* < 0.05 was considered statistically significant. Triangles, diamonds, circles, and squares are used solely to differentiate between experimental groups and do not indicate specific biological meanings. Each dot represents data from an independent experiment.

To investigate the synergistic effect of *I. latifolia*, *L. robustum*, and rapamycin, we used a concentration of 0.25 mg/mL for both *I. latifolia* and *L. robustum*, which individually did not influence the growth of breast cancer cells (Figure 1). These were amalgamated with the minimal effective dosage of rapamycin (8 μM, Figure 1). Indeed, neither 0.25 mg/mL *I. latifolia* nor *L. robustum* alone could inhibit breast tumor growth (Figure 2A). Interestingly, *I. latifolia* exhibited potent activity in enhancing the antiproliferative effects of rapamycin (Figure 2A). In contrast, combining *L. robustum* with rapamycin did not enhance the inhibitory effect on cell proliferation observed with rapamycin alone (Figure 2A). Additionally, the CCK-8 assay demonstrated that rapamycin induced tumor cell death (Figure 2B). When combined with *I. latifolia*, cell death was further increased in all four breast cancer cell lines (Figure 2B). However, the combination of *L. robustum* with rapamycin did not amplify the anti-tumor effects of rapamycin (Figure 2B). These findings suggest that *I. latifolia* may serve as an effective dietary adjunct to rapamycin for enhancing its anti-tumor activity, while *L. robustum* does not provide the same synergistic effect.

### 3.4. Ilex latifolia Enhances Rapamycin-Induced Tumor Cell Cycle Arrest

**Figure 3:**
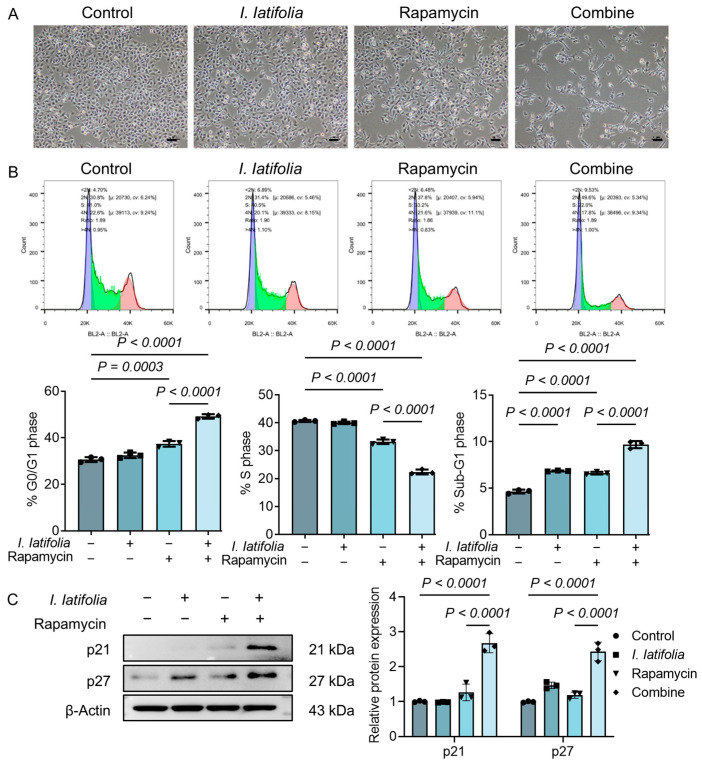
Effects of *Ilex latifolia* combined with rapamycin on the cell cycle. (**A**) Representative images of cell morphology under a microscope (scale bar: 50 μm). (**B**) Flow cytometry analysis of cell cycle distribution. (**C**) Protein expression and quantification of p21 and p27 in HCC1806 cells. Data in (**B**,**C**) were presented as mean ± SD from three independent experiments. *p* values in (**B**,**C**) were determined by Tukey’s multiple comparisons test following one-way ANOVA. *p* < 0.05 was considered statistically significant. Triangles, diamonds, circles, and squares are used solely to differentiate between experimental groups and do not indicate specific biological meanings. Each dot represents data from an independent experiment.

To further investigate the adjuvant role of *I. latifolia* in rapamycin treatment, HCC1806 cells were selected based on their pronounced response in Figure 2. Morphological changes under different treatments were visualized using microscopy. Under normal conditions, HCC1806 cells grew in clusters and adhered tightly to the culture dish. Low concentrations of *I. latifolia* extract or rapamycin alone did not significantly alter cell morphology. However, the combined treatment caused notable morphological alterations at the cell edges, with a significant reduction in cell number (Figure 3A). To evaluate the influence on the cell cycle, flow cytometry analysis was performed. Cells in the G0/G1 phase (2n), S phase (2n~4n), and sub-G1 phase (<2n) were observed, with the G0/G1 phase linked to cell quiescence, the S phase representing DNA replication, and the sub-G1 peak indicating apoptotic cell populations. Rapamycin promoted the G0/G1 phase, inhibited the S phase, and increased the sub-G1 phase (Figure 3B). This indicates that rapamycin therapy activates cellular apoptosis mechanisms and disrupts normal cell cycle progression. Interestingly, the combination of rapamycin and *I. latifolia* further enhances rapamycin-induced tumor cell cycle progression disruption (Figure 3B). Additionally, Western blotting analysis revealed elevated expression levels of P21 and P27 proteins following combination treatment (Figure 3C). These results suggest that *I. latifolia* enhances rapamycin-induced cell cycle arrest, particularly at the G1 phase, thereby inhibiting tumor cell proliferation.

### 3.5. Ilex latifolia Augments Rapamycin-Induced Modulation of Apoptosis and Inflammation in Breast Cancer Cells

**Figure 4:**
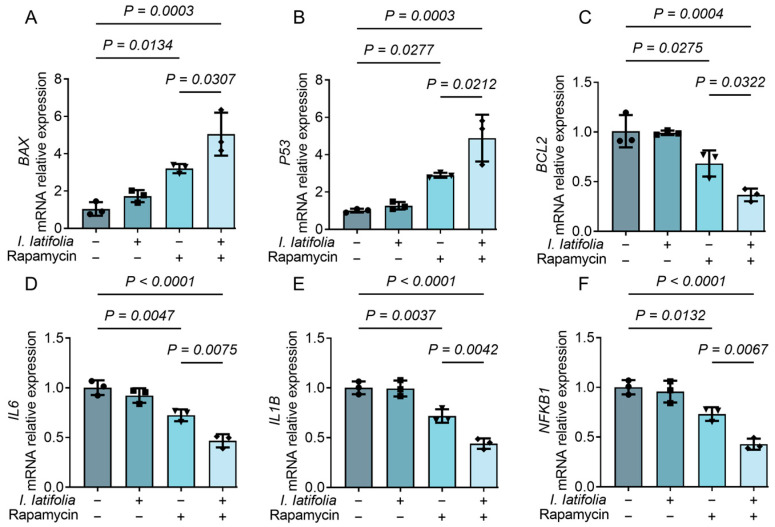
Effects of *Ilex latifolia* combined with rapamycin on apoptosis and inflammation. (**A**–**C**) Relative expression levels of apoptosis-related genes (**A**) *BAX*, (**B**) *P53*, and (**C**) *BCL2*. (**D**–**E**) Relative expression levels of inflammation-related genes (**D**) *IL6*, (**E**) *IL1B*, and (**F**) *NFKB1*. Data in (**A**–**F**) were presented as mean ± SD from three independent experiments. *p* values in (**A**–**F**) were determined by Tukey’s multiple comparisons test following one-way ANOVA. *p* < 0.05 was considered statistically significant. Triangles, diamonds, circles, and squares are used solely to differentiate between experimental groups and do not indicate specific biological meanings. Each dot represents data from an independent experiment.

To investigate the effects of *I. latifolia* and rapamycin combination treatment, we examined the expression of apoptosis-related genes (*BAX*, *BCL2*, and *P53*) and inflammation-related genes (*IL6*, *IL1B*, and *NFKB1*) in HCC1806 cells using RT-qPCR. The results revealed that in comparison to the control group, combination treatment with *I. latifolia* and rapamycin significantly upregulated the expression of *BAX* and *P53*, both of which are associated with apoptosis induction (Figure 4A,B). On the other hand, the anti-apoptotic protein *BCL2* was downregulated following the combined treatment (Figure 4C). In terms of inflammatory factors, *IL6*, *IL1B*, and *NFKB1* expression levels were notably reduced in the combination treatment group relative to the control group, suggesting a potential reduction in tumor-associated inflammation (Figure 4D–F). Notably, the combination treatment group showed more significant effects than rapamycin treatment alone (Figure 4A–F). These findings indicate that the combination of *I. latifolia* and rapamycin enhances apoptosis and modulates inflammatory responses in HCC1806 cells.

### 3.6. Combined Treatment of Ilex latifolia and Rapamycin Inhibits Breast Cancer Cell Growth in Mice

**Figure 5:**
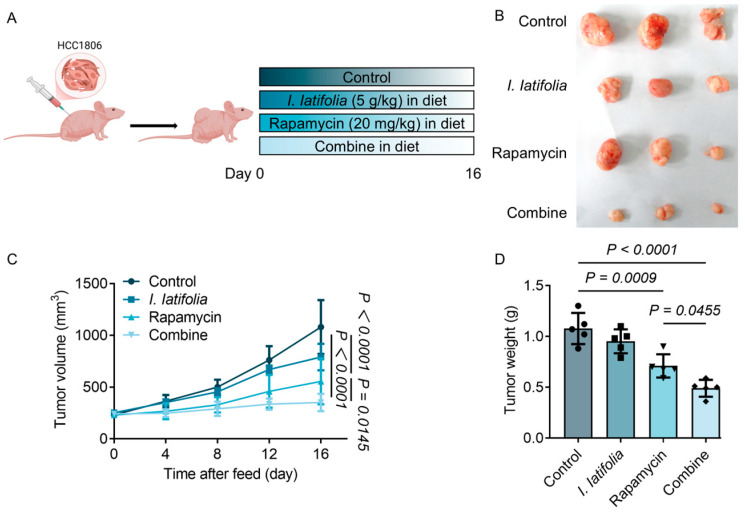
The protective effect of *Ilex latifolia* combined with rapamycin on breast cancer mice. (**A**) Animal protocol (diagram created with BioRender.com). (**B**) Representative photos of tumors. (**C**) Tumor volume changes (*n* = 5). (**D**) Tumor weight (*n* = 5). Data in (**C**) were presented as mean and error ± SD and *p* values were determined by Tukey’s multiple comparisons test following two-way ANOVA. Data in (**D**) were presented as mean ± SD and *p* values were determined by Tukey’s multiple comparisons test following one-way ANOVA. *p* < 0.05 was considered statistically significant. Triangles, diamonds, circles, and squares are used solely to differentiate between experimental groups and do not indicate specific biological meanings. Each dot represents data from an individual mouse.

After confirming the synergistic effect of *I. latifolia* and rapamycin in vitro, we proceeded to further investigate their therapeutic potential in vivo. Mice were randomly allocated to the designated treatment groups, and tumors were surgically removed after the treatment period for analysis (Figure 5A). Visual inspection of the excised tumors revealed notable differences in tumor size across the groups. The combined treatment group showed significantly smaller tumors compared to both the control and single-treatment groups, suggesting a more pronounced anti-tumor effect when *I. latifolia* and rapamycin were used together (Figure 5B). Tumor growth was closely monitored over time, as shown in Figure 5C. Before the initiation of the treatment, no notable disparities in tumor volume were detected among the groups, indicating that the tumors grew at a similar rate. However, as the treatment progressed, the control group had a substantial augmentation in tumor volume, while the tumors in the treatment groups showed slower growth. By day 16, tumors in the rapamycin-treated group were significantly smaller and lighter compared to the control, confirming its effective inhibition of tumor growth (Figure 5C,D). Notably, the combined treatment with *I. latifolia* further reduced tumor volume and weight, augmenting the anti-neoplastic properties of rapamycin (Figure 5C,D). These results indicate that *I. latifolia* enhances the anti-tumor efficacy of rapamycin in vivo.

### 3.7. Ilex latifolia Enhances the Anti-Tumor Effect of Rapamycin in Mice

**Figure 6:**
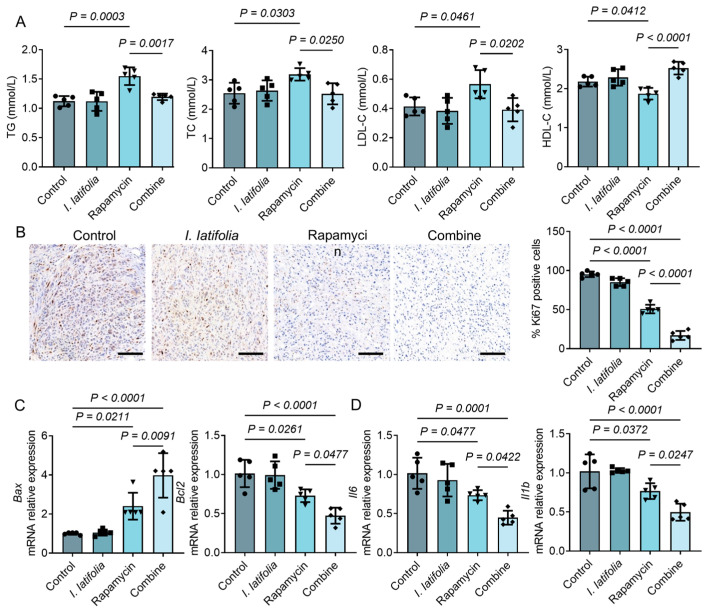
Effect of combined *Ilex latifolia* and rapamycin treatment on breast cancer mice. (**A**) Blood levels of triglycerides (TG), total cholesterol (TC), low-density lipoprotein cholesterol (LDL-C), and high-density lipoprotein cholesterol (HDL-C) in mice (*n* = 5). (**B**) Quantification of Ki-67 staining in tumor tissues (*n* = 5, scale bar: 100 μm). (**C**) Relative expression of apoptosis-related genes *Bax* and *Bcl2* in tumor tissues (*n* = 5). (**D**) Relative mRNA expression of inflammation cytokines *Il6* and *Il1b* in tumor tissues (*n* = 5). Data in (**A**–**D**) were presented as mean ± SD from three independent experiments. *p* values in (**A**–**D**) were determined by Tukey’s multiple comparisons test following one-way ANOVA. *p* < 0.05 was considered statistically significant. Triangles, diamonds, circles, and squares are used solely to differentiate between experimental groups and do not indicate specific biological meanings. Each dot represents data from an individual mouse.

We further investigated the effects of combining *I. latifolia* with rapamycin on lipid metabolism, tumor cell proliferation, apoptosis, and inflammation. Our results showed that *I. latifolia* significantly improved the lipid abnormalities induced by rapamycin, including reductions in triglycerides (TG), total cholesterol (TC), and low-density lipoprotein cholesterol (LDL-C), and increasing high-density lipoprotein cholesterol (HDL-C) (Figure 6A). These findings suggest that *I. latifolia* can alleviate the lipid-related side effects of rapamycin. Additionally, we evaluated tumor cell proliferation using immunohistochemistry to detect Ki67 expression, a marker of proliferative activity. Ki67-positive cells, stained dark brown to black, were markedly diminished in the combination therapy group relative to the control and single-treatment groups (Figure 6B). Quantitative analysis confirmed that the number of Ki67-positive cells in the combined group was significantly lower than in the other groups (Figure 6B). These results demonstrate that the combination of *I. latifolia* and rapamycin effectively inhibits tumor cell proliferation. Moreover, the expression of genes associated with apoptosis (*Bax* and *Bcl2*) and inflammation (*Il6* and *IL1b*) in tumor tissues was notably modulated by rapamycin (Figure 6C,D). Dietary treatment with *I. latifolia* augmented the pro-apoptotic and anti-inflammatory effects of rapamycin in breast cancer mice. These data highlight the efficacy of this combination medication in improving breast cancer treatment.

## 4. Discussion

This study demonstrates that the combination of low-dose rapamycin with *I. latifolia* represents a promising strategy to enhance breast cancer therapy while mitigating rapamycin-induced lipid metabolic disorders. Although low-dose *I. latifolia* alone lacked significant anti-tumor activity, its synergistic effects with low-dose rapamycin were evident both in vitro and in vivo. This combination not only inhibited tumor proliferation, induced apoptosis, and suppressed inflammation but also alleviated rapamycin-associated metabolic side effects. These findings underscore the potential of *I. latifolia* as a dietary supplement to complement rapamycin-based therapies, offering a safer and more effective approach to breast cancer treatment.

Rapamycin, an mTOR inhibitor, has demonstrated promising efficacy in breast cancer therapy by focusing on critical mechanisms implicated in tumor growth and persistence [7,25]. Both preclinical and clinical investigations demonstrate that rapamycin inhibits tumor growth by blocking mTOR-driven cell cycle progression and promoting apoptosis [26]. This is achieved through the downregulation of anti-apoptotic proteins, such as Bcl-2, and the upregulation of pro-apoptotic factors, such as Bax [27]. A meta-analysis of clinical trials demonstrated that rapamycin-based regimens significantly extended progression-free survival in individuals with advanced or treatment-resistant breast carcinoma. However, these benefits were associated with notable side effects, including hyperlipidemia, metabolic disturbances, and immunosuppression [28]. These toxicities limit the long-term use of rapamycin, highlighting the need for strategies to mitigate adverse reactions while improving therapeutic efficacy. Interestingly, natural compounds may serve as effective adjuncts to rapamycin. For example, compounds like resveratrol and berberine have been shown to enhance autophagic flux when combined with rapamycin, potentially overcoming drug resistance in breast cancer models [29,30]. However, clinical validation is necessary to confirm these interactions in human subjects.

*I. latifolia*, commonly known as large-leaf Kudingcha, is a traditional Chinese tea with over 2000 years of history [13]. Predominantly cultivated in the Hainan and Zhejiang provinces, it is recognized for its unique bioactive components and health-promoting properties [31]. Our analysis identified 1,4-dicaffeoylquinic acid, 1-caffeoylquinic acid, esculentoside L, and ruberythric acid as major bioactive components in the extract of *I. latifolia*. These compounds are known for various biological activities and may contribute to the enhanced anti-tumor effects observed in combination with low-dose rapamycin. Given that our study observed reduced inflammatory markers and increased apoptosis following co-treatment, it is plausible that chlorogenic acid derivatives, especially 1,4-dicaffeoylquinic acid, contribute to these effects via suppression of the NF-κB pathway and modulation of pro-apoptotic signaling. These mechanisms are consistent with previous reports and may underlie the enhanced anti-tumor activity observed in the *I. latifolia* and rapamycin combination [32,33,34]. Additionally, esculentoside L, a triterpenoid saponin, has been reported to exert anti-inflammatory and pro-apoptotic effects in several diseases, including cancer [35]. Ruberythric acid, an anthraquinone compound, has demonstrated cytotoxic and anti-inflammatory properties [36]. These bioactive constituents likely work together to give *I. latifolia* its anti-breast cancer activity. However, the bitterness of *I. latifolia* limits its widespread acceptance. Incorporating it at minimal effective doses into daily diets as an adjuvant to pharmacological treatments could offer a feasible strategy to enhance therapeutic efficacy while maintaining patient compliance.

Dietary intervention has emerged as a promising strategy in the prevention and management of breast carcinoma, focusing on both dietary patterns and specific functional foods with potential anti-cancer properties [37,38]. Clinical approaches emphasize the role of nutrition in modulating the molecular pathways involved in cancer development, including inflammation and oxidative stress [39]. Dietary interventions incorporating antioxidant-rich foods, such as fruits, vegetables, and whole grains, have demonstrated a reduction in breast carcinoma risk by mitigating oxidative damage and enhancing immune function [40,41]. Additionally, specific bioactive compounds found in foods, such as polyphenols, flavonoids, and omega-3 fatty acids, have demonstrated anti-inflammatory and anti-cancer effects by targeting key signaling pathways like NF-κB [42,43,44]. In this context, *I. latifolia* represents a promising dietary adjunct to rapamycin, offering both anti-tumor and lipid-regulating benefits. However, its optimal dose range, bioavailability, and long-term safety remain to be fully elucidated. Future food-based interventions should consider these factors, along with personalized nutrition strategies, to maximize therapeutic efficacy.

This study reveals that *I. latifolia* can potentiate the anti-tumor efficacy of low-dose rapamycin, as evidenced by the observed inhibition of tumor proliferation, promotion of apoptosis, attenuation of inflammation, and reduction in tumor burden in both in vitro and in vivo models. While *I. latifolia* alone exhibited limited efficacy, its combination with rapamycin produced synergistic effects and alleviated rapamycin-induced metabolic disturbances. These findings suggest that *I. latifolia* may serve as a potential dietary adjuvant to enhance the therapeutic benefit of rapamycin-based regimens, warranting further investigation into its translational relevance.

## Figures and Tables

**Table 1:** Sequences of primers utilized in this research.

Gene	Species	Forward	Reverse
*BAX*	Human	5′-CCCGAGAGGTCTTTTTCCGAG-3′	5′-CCAGCCCATGATGGTTCTGAT-3′
*BCL2*	Human	5′-GGTGGGGTCATGTGTGTGG-3′	5′-CGGTTCAGGTACTCAGTCATCC-3′
*P53*	Human	5′-CAGCACATGACGGAGGTTGT-3′	5′-TCATCCAAATACTCCACACGC-3′
*IL6*	Human	5′-ACTCACCTCTTCAGAACGAATTG-3′	5′-CCATCTTTGGAAGGTTCAGGTTG-3′
*IL1B*	Human	5′-ATGATGGCTTATTACAGTGGCAA-3′	5′-GTCGGAGATTCGTAGCTGGA-3′
*NFKB1*	Human	5′-AACAGAGAGGATTTCGTTTCCG-3′	5′-TTTGACCTGAGGGTAAGACTTCT-3′
*ACTB*	Human	5′-CATGTACGTTGCTATCCAGGC-3′	5′-CTCCTTAATGTCACGCACGAT-3′
*Bax*	Mouse	5′-TGAAGACAGGGGCCTTTTTG-3′	5′-AATTCGCCGGAGACACTCG-3′
*Bcl2*	Mouse	5′-GTCGCTACCGTCGTGACTTC-3′	5′-CAGACATGCACCTACCCAGC-3′
*Il6*	Mouse	5′-TAGTCCTTCCTACCCCAATTTCC-3′	5′-TTGGTCCTTAGCCACTCCTTC-3′
*Il1b*	Mouse	5′-GCAACTGTTCCTGAACTCAACT-3′	5′-ATCTTTTGGGGTCCGTCAACT-3′
*Actb*	Mouse	5′-AATCCCATCACCATCTTCCA-3′	5′-TGGACTCCACGACGTACTCA-3′

**Table 2:** Composition of *Ilex latifolia*.

Constituents Class	Representative Subclass	Relative Content (%)
Saponins	Triterpenoid saponins	46.89349
Polyphenols	Phenolic acids	45.96911
Alkaloids	Pyrrolizidine alkaloids	2.914044
Terpenoids	Sesquiterpenes	1.920293
Organic acids	Citric acid derivatives	1.270804
Lipids	Glyceride	0.630561
Aromatic compounds	Benzene compounds	0.170388
Carbohydrates	Polysaccharides	0.117496
Steroids	Phytosterols	0.04531
Glycosides	Cyanogenic glycosides	0.03653
Amino acids	Essential amino acids	0.016108
Ketones	Quinones	0.009137
Alcohols	Aliphatic Alcohols	0.006725

**Table 3:** Top 10 predominant components in *Ilex latifolia*.

No	Component Name	t_R_ (min)	Formula	Molecular Ion (*m*/*z*)	Peak Area	Ion Mode	Relative Content (%)
1	1,4-dicaffeoylquinic acid	7.41	C_25_H_24_O_12_	515.1198	13,226,556	[M−H]^−^	17.17072603
2	1-caffeoylquinic acid	5.08	C_16_H_18_O_9_	353.0884	8,470,651	[M−H]^−^	10.99660619
3	esculentoside L	11.70	C_48_H_76_O_20_	971.4829	6,761,731	[M−H]^−^	8.778084822
4	ruberythric acid	6.08	C_25_H_26_O_13_	533.1303	4,749,673	[M−H]^−^	6.166029449
5	cynanoside O	12.82	C_48_H_74_O_19_	953.4716	4,566,480	[M−H]^−^	5.928208144
6	marsdenoside H	13.73	C_48_H_76_O_19_	955.4881	3,383,373	[M−H]^−^	4.39229765
7	clinodiside A	12.04	C_48_H_78_O_19_	957.5033	2,835,076	[M−H]^−^	3.680498027
8	clinodiside B	13.41	C_54_H_88_O_23_	1103.5600	2,428,726	[M−H]^−^	3.152974118
9	nelumboroside A	6.83	C_27_H_30_O_16_	609.1455	1,692,296	[M−H]^−^	2.196940078
10	soyasaponin I	14.33	C_48_H_78_O_18_	941.5083	1,652,682	[M−H]^−^	2.14551315

## Data Availability

The original contributions presented in this study are included in the article. Further inquiries can be directed to the corresponding author.

## Abbreviations

The following abbreviations are used in this manuscript:

ANOVA	One-way analysis of variance
ER	Estrogen receptor
HDL-C	High-density lipoprotein cholesterol
HER2	Human epidermal growth factor receptor 2
*I. latifolia*	*Ilex latifolia*
LDL-C	Low-density lipoprotein cholesterol
*L. robustum*	*Ligustrum robustum*
mTOR	Mammalian target of rapamycin
PR	Progesterone receptor
SD	Standard deviation
TC	Total cholesterol
TG	Triglycerides
TNBC	Triple-negative breast cancer
